# The impact of blastomere loss on pregnancy and neonatal outcomes of vitrified-warmed Day3 embryos in single embryo transfer cycles

**DOI:** 10.1186/s13048-022-00997-z

**Published:** 2022-05-18

**Authors:** Shutian Jiang, Wei Jin, Xinxi Zhao, Qianwen Xi, Li Chen, Yining Gao, Wenzhi Li, Yanping Kuang

**Affiliations:** Department of Assisted Reproduction, Shanghai Ninth People’s Hospital, Shanghai Jiaotong University School of Medicine, 639 Zhizaoju Road, Shanghai, 200011 China

**Keywords:** Blastomere loss, Pregnancy outcomes, Neonatal outcomes, Vitrification, Single frozen embryo transfer

## Abstract

**Background:**

Blastomere loss is a common phenomenon that occurs following cryopreservation. To date, studies have drawn conflicting conclusions regarding the impact of blastomere loss on pregnancy outcomes. Besides, limited information is available concerning the neonatal safety of embryos with blastomere loss. In the present study, we aimed to investigate the impact of blastomere loss on pregnancy and neonatal outcomes of vitrified/warmed Day3 cleavage-stage embryos in single embryo transfer cycles.

**Methods:**

This retrospective cohort study included all vitrified/warmed D3 cleavage-stage single frozen-thawed embryo transfer (FET) cycles between April 2015 and February 2021. We compared pregnancy and subsequent neonatal outcomes between the intact embryos group and the blastomere loss group in single FET cycles.

**Results:**

A total of 6287 single FET cycles were included in the study, in which 5873 cycles were classified into the intact embryo group and 414 cycles were classified into the blastomere loss group. The outcomes of the blastomere loss group were significantly inferior to those of the intact embryo group, in terms of implantation/biochemical pregnancy/clinical pregnancy/ongoing pregnancy rate and live birth rate per embryo transfer cycle/per clinical pregnancy. Further binary logistic regression confirmed that blastomere loss was negatively associated with live birth. Moreover, the blastomere loss group presented with an elevated early miscarriage rate. The neonatal conditions were broadly similar between the two groups. Additionally, multiple binary logistic regression analysis demonstrated that primary infertility and intracytoplasmic sperm injection (ICSI) were common influencing factors of blastomere loss (aOR 1.447, 95% CI 1.038–2.019, *P* = 0.029; aOR: 1.388, 95% CI: 1.044–51.846, *P* = 0.024).

**Conclusions:**

The transfer of vitrified/warmed D3 embryos with blastomere loss is related to impaired embryo developmental potentials and reduced probabilities of conception. Moreover, even if the embryos with blastomere loss have implanted and reached clinical pregnancies, they present with a lower possibility of developing to live birth owing to a higher early miscarriage rate. However, once the embryos with blastomere loss result in a live birth, no adverse neonatal outcomes are observed.

Primary infertility and ICSI were found to be risk factors for blastomere loss.

**Supplementary Information:**

The online version contains supplementary material available at 10.1186/s13048-022-00997-z.

## Introduction

Since the first live birth of a human being resulting from frozen embryo transfer (FET) in 1984 [1], cryopreservation has become more and more widely adopted and gradually occupied the mainstream position in the field of assisted reproduction technology (ART) [2]. Embryo cryopreservation with subsequent FET not only exhibits its advantage in increasing the cumulative pregnancy rate per oocyte retrieval cycle, but also contributes to the decreased risk of multiple pregnancies [3]. Besides, FET is beneficial to prevent the occurrence of ovarian hyperstimulation syndrome (OHSS) [4].

While embryo cryopreservation has become an essential part of treatment with ART, the comparison of two routine embryo cryopreservation methods, namely slow freezing and vitrification, has drawn a great deal of attention [5]. Recently, a progressive switch from slow freezing to vitrification has emerged, given the significantly improved survival rate as well as higher implantation, pregnancy and live birth rate of the latter method [6].

Despite the extensive development, a series of problems with the technique are still unresolved, and many aspects remain to be studied [7]. Blastomere loss, a phenomenon that occurs following cryopreservation and thawing of embryos, has captured the focus of researchers. It is usually perceived as one of the parameters to evaluate the implantation potential of frozen-thawed embryos. To date, conflicting results have been yielded by a variety of studies regarding the influence of blastomere loss on pregnancy and perinatal outcomes of cleavage-stage embryos. Some studies have demonstrated that blastomere loss is not related to a decreased implantation rate [8–10], even if the conclusion is sometimes conditional (i.e., only embryos that resumed mitotic activity after thawing were transferred) [11, 12]. However, other studies have revealed a deleterious effect of blastomere loss on the developmental capacity of embryos, including the in vitro process of mitosis or blastocyst formation and the post-transfer ability of conception [13–16]. Of note, there are also several studies that reported negative influences of blastomere loss in terms of some pregnancy-related indicators, while claiming blastomere loss as harmless regarding the other indices [17–19].

The inconsistency of the results so far could be ascribed to the limitations of previous studies and the variability within them. First, the cryopreservation method differs between studies and some studies may recruit both slowing freezing and vitrification. Taking into account the different characteristics of the two methods, it is obvious that the proportion will affect the outcomes. Second, most studies contain both multiple embryo transfer and single embryo transfer procedures. Considering the probable unequal damage extent of the embryos and the mutual influence between embryos in a multiple embryo transfer cycle, it is might inappropriate to mix the two procedures in one study when comparing. Even if the above points can be overcome by separating one of the methods/procedures for analysis, it will not be able to draw a definitive conclusion due to the reduced sample size. Third, some of the results are obtained in the cases of Day 2 embryo, while others are produced in the cases of Day 3 embryo. This discrepancy in the setting will lead to the discordance of the results. Moreover, as time goes by, the innovation in technology and culture medium is likely to have a potential beneficial effect on the endpoints of observations.

Therefore, in this retrospective study, we aimed to investigate the effect of the blastomere loss on pregnancy outcomes and neonatal conditions, compared with those of intact embryos. The impact was analyzed in the setting of single FET of cleavage-stage embryos, in which all embryos were cryopreservation by vitrification on Day 3.

## Methods

### Study settings and patients

This retrospective cohort study was carried out at the Department of Assisted Reproduction of the Ninth People’s Hospital affiliated with Shanghai Jiao Tong University School of Medicine. Cases of patients undergoing FET between April 2015 and February 2021 were recruited. Inclusion criteria included age < 42 years old, and BMI within 18–28 kg/m^2^. Meanwhile, only single FET cycles with embryos vitrified and transferred on Day 3 at the cleavage-stage were included in this study. Cycles with donated gametes or preimplantation genetic diagnosis (PGD) or originating from in vitro maturation were excluded. Furthermore, for patients who experienced twice FET during the period, only data from the first cycle were included. When core data were missing, the corresponding cycle was also excluded. The study was approved by the hospital’s ethics committee. The follow-up system has been described in detail in our previous studies (Du et al., 2017; Huang et al., 2019). The final data, involving 6287 FET cycles in total, were divided into two groups: 5873 intact embryo cycles and 414 blastomere loss cycles. The reporting of this study conforms to the STROBE statement.

### Procedures

#### Ovarian stimulation and IVF/ICSI protocols

In the beginning, the sociodemographic characteristics and reproductive history of the women and their partners were inquired and documented. Then after controlled ovarian stimulation, oocyte retrieval was performed around 36 h after the trigger, once at least one dominant follicle reached 20 mm in diameter, or three dominant follicles reached 18 mm in diameter. Depending on the parameters of semens, either conventional insemination or intracytoplasmic sperm injection (ICSI) was adopted for fertilization 4–6 h after oocyte retrieval, the procedure of which has been detailed previously [20]. Generally, IVF was performed in human tubal fluid (HTF; Irvine Scientific, Santa Ana, CA, USA) with 10% (v/v) serum substitute supplement (SSS; Irvine Scientific, Santa Ana, CA, USA) and ICSI was conducted in separate microdroplets containing HTFt10% SSS. The fertilization was assessed 16–.

20 h later. All the embryos were cultured in a humidified atmosphere containing 5% O2 and 6% CO2 at 37 °C in the Continuous Single Culture medium (Irvine Scientific, Santa Ana, CA, USA). The laboratory procedures and materials remained unchanged throughout the study period.

#### Embryo freezing and thawing protocols

All embryos included in this study were cryopreserved at D3 after insemination by vitrification, due to either a maternal condition that was unsuitable for fresh embryo transfer, such as a high risk of ovarian hyperstimulation syndrome, a desynchronized endometrium, or when supernumerary embryos had been obtained in a previous oocyte retrieval cycle. Examinations of the number of blastomeres were performed and scores for the embryos were given according to Cummins’s criteria, prior to freezing. The detailed procedures of vitrification and thawing were described previously [20]. Briefly, Embryos were vitrified using a Cryotop carrier system (Kitazato Biopharma Co., Shizuoka, Japan) with a mixture of dimethyl sulfoxide, ethylene glycol and sucrose as cryoprotectants. Embryo thawing was performed in a descending concentration gradient of sucrose (1–0.5–0 mol/L). All vitrification and thawing steps were carried out at room temperature except for the first warming step, which was conducted at 37 °C.

Embryos were evaluated morphologically immediately after thawing for the blastomere number.

#### Endometrial preparation for single FET

Before embryo thawing, endometria were prepared for FET by natural cycles, ovarian stimulated cycles, or hormone replacement cycles for patients with regular ovulatory cycles, irregular menses, or a history of thin endometrium, respectively, as we previously reported [20, 21]. Embryos were thawed the same day as embryo transfer and replaced under ultrasound guidance as a Day 3 cleavage-stage embryo on the third day after progesterone treatment. In general, embryos losing more than 50% of their original blastomere are not suitable for transfer, so only embryos with at least 50% of their blastomeres survived would be transferred in our study [14]. When pregnancy was achieved, the luteal support was continued until 12 weeks of gestation.

#### Follow-up of pregnancy and neonatal outcomes

Pregnancy outcome measures following FET were assessed at follow-up visits. Ultrasound scans and the serum β-hCG level were part of the routine clinical care. The newborn follow-up system at our department has been established for years and depicted previously [22, 23]. In brief, the couples completed a total of 4 telephone surveys by trained nurses during each trimester of pregnancy and up to 4 weeks after delivery. Standardized questionnaires were used to collect the following information: pregnancy exposures, pregnancy complications, gestational weeks, mode of delivery, birth date and locality, birth weight and length, newborn gender, neonatal diseases, and congenital defects. For babies born in our university hospital, the medical records were obtained through the electronic network system, while written proof was acquired from the pediatrician in charge of the babies were born elsewhere. Furthermore, for newborns with congenital defects, a particular nurse was designated for a thorough review to guarantee their accordance with the case definition of the Chinese Birth Defects Monitoring Program.

#### Outcome measures and definitions

Pregnancy outcome assessments following FET cycles included the implantation rate, biochemical pregnancy rate, clinical pregnancy rate, ongoing pregnancy rate, live birth rate per transfer cycle, and live birth per clinical pregnancy. The implantation rate was defined as the number of gestational sacs measured by ultrasound relative to the number of embryos transferred. The biochemical pregnancy rate denoted the proportion of women with a serum βhCG concentration > 5 mIU/ml collected 14 days after FET. Clinical pregnancy was confirmed by the ultrasonic demonstration of an intrauterine gestational sac at 6–8 gestational weeks, and the clinical pregnancy rate was calculated using the number of clinical pregnancies divided by the number of FET cycles. Ongoing pregnancy was referred to a pregnancy with a fetal heartbeat at 12 weeks of gestational age. The ongoing pregnancy rate was calculated by dividing the number of ongoing pregnancies by the number of FET cycles. Live birth was defined as the delivery of one or more infants with any signs of life after 28 weeks of gestation, and the live birth rate was calculated on the basis of the number of FET cycles and clinical pregnancies. Moreover, adverse outcomes included early miscarriage before 12 gestational weeks, second trimester abortions12–28 gestational weeks, stillbirth, and ectopic pregnancy. Biochemical pregnancy loss was defined as a positive pregnancy test in the absence of any ultrasonographic evidence of pregnancy, and no evidence or treatment of an extrauterine pregnancy. Miscarriage was defined as a pregnancy with spontaneous termination of pregnancy. Ectopic pregnancy was defined as the implantation of an embryo outside the uterine cavity. Stillbirth was defined as the delivery of a baby born without signs of life after 28 gestational weeks.

The neonatal outcome measures included gestational age (defined as Day 17 for cleavage-stage embryo transfer), newborn gender, birthweight, birth length, congenital defects, and neonatal mortality. Small for gestational age (SGA) was defined as a birthweight below the 10th percentile for the gestational age, and large for gestational age was defined as a birthweight over the 90th percentile [24]. A congenital defect was defined as a deformity and/or developmental abnormality of any organ or system. Neonatal mortality referred to infant death during the first 28 days of life. Z-scores were calculated after adjusting for the gestational age and the newborn gender, birthweight percentile, and Z-scores were calculated based on a general population reference for Chinese singletons [25].

### Statistical analysis

Statistical analyses were performed using SPSS 19.0 software (SPSS, Inc.). Data were presented as means±SD if they demonstrated normal distributions, or as medians (quartiles) for non-normal distributions; Qualitative data were put forward in percentages. The normality was tested by the Shapiro–Wilk test and the homogeneity of variances was tested by Levene’s test. Different kinds of continuous parametric data were analyzed by different means: Student’s t-test was used to compare the means and the Mann-Whitney U test was used to compare the medians. The rates were compared between groups by the chi-square test or Fisher’s exact test as appropriate. Binary logistic regression was performed to quantify the influential factors on blastomere loss and live birth, in which an adjusted odds ratio (aOR) and 95% confidence interval (CI) were displayed. *P* < 0.05 was considered statistically significant.

## Results

A total of 6287 single FET cycles of cleavage-stage embryos from 6287 patients were included from April 2015 and February 2021 in this study. Among them, 5873 cycles were sorted into the intact embryo group, and the remaining 414 cycles were classified as the blastomere group. All of the patients recruited in the present study completed the follow-up until 28 days after delivery and the live-born singletons were included in the neonatal outcomes analysis.

As presented in Table 1, most of the demographic and basic characteristics were comparable between the two groups, including maternal age, parental age, maternal body mass index (BMI), duration of infertility, the proportion of multiparity, distribution of endometrium preparation protocols, endometrial thickness and the basal sexual hormone profiles. Patients with primary infertility were less frequent in the intact embryo group than in the blastomere loss group (51.00% vs. 58.70%, *p* = 0.003). Moreover, differences could be observed in the distribution of the insemination method that embryos originated from between the two groups. The percentage of IVF in the intact embryo group was obviously higher than that of the blastomere loss group (64.53% vs. 55.56%, *p* < 0.001), while the proportion of ICSI in the intact embryo group was clearly lower.

**Table 1:** Baseline characteristics of all transfer cycles

	Intact embryo group (*n* = 5873)	Blastomere loss group (*n* = 414)	*P*
Maternal age (years)	34.88 ± 5.57	34.43 ± 5.53	0.253
Parental age (years)	36.46 ± 6.47	36.06 ± 6.26	0.383
Maternal BMI (kg/m^2^)	22.27 ± 5.88	22.01 ± 3.22	0.262
Duration of infertility (years)	3.22 ± 3.19	3.43 ± 3.00	0.344
Primary infertility (n,%)	2995/5873 (51.00%)	243/414 (58.70%)	0.003*
Pluriparous (n,%)	979/5873 (16.67%)	63/414 (15.22%)	0.494
Type of FET cycles			0.074
Natrual	836/5873 (14.23%)	51/414 (12.32%)	
HRT	2562/5873 (43.62%)	165/414 (39.86%)	
OS	2475/5873 (42.14%)	198/414 (47.83%)	
Insemination method of embryos			< 0.001*
IVF	3790/5873 (64.53%)	230/414 (55.56%)	
ICSI	2083/5873 (35.47%)	184/414 (44.44%)	
Endometrial thickness (mm)	10.42 ± 2.38	10.67 ± 2.56	0.125
Basal FSH (mIU/ml)	5.86 (4.81–7.72)	5.54 (4.53–7.18)	0.052
Basal LH (mIU/ml)	4.48 ± 4.25	4.47 ± 3.76	0.974
Basal E2 (pg/ml)	47.33 ± 47.13	44.11 ± 35.79	0.355
Basal P4 (ng/ml)	0.20 (0.20–0.30)	0.20 (0.20–0.30)	0.526

Pregnancy outcomes following single FET are shown in Table 2. The implantation rate was notably higher in the intact embryo group than in the blastomere loss group (31.56% vs. 21.01%, *p* < 0.001). The intact embryo group displayed obviously higher rates in terms of the biochemical pregnancy rate (34.97% vs. 24.64%, *p* < 0.001), clinical pregnancy rate (30.67% vs. 21.01%, p < 0.001), ongoing pregnancy rate (25.63% vs. 15.70%, p < 0.001), live birth rate per embryo transfer cycle (24.92% vs. 14.25%, p < 0.001), and live birth rate per clinical pregnancy (81.23% vs. 67.82%, *p* = 0.003), compared with the blastomere loss group. Moreover, the early miscarriage rate was significantly lower in the intact embryo group than in the blastomere loss group (16.44% vs. 25.29%, *p* = 0.039). Nonetheless, the two groups were comparable regarding the biochemical pregnancy loss rate, ectopic pregnancy rate, second-trimester abortion rate, stillbirth rate, and the proportion of singleton pregnancy. In addition, the monozygotic twin rate was 1.78 (26/1463) in the intact embryo group, while no case of monozygotic twin was observed in the blastomere loss group.

**Table 2:** Pregnancy outcomes following transferring embryos with or without blastomere loss

	Intact embryo group (*n* = 5873)	Blastomere loss group (*n* = 414)	*P*
Implantation rate (n,%)	1853/5873 (31.56%)	87/414 (21.01%)	< 0.001
Biochemical pregnancies (n,%)	2054/5873 (34.97%)	102/414 (24.64%)	< 0.001
Clinical pregnancies (n,%)	1801/5873 (30.67%)	87/414 (21.01%)	< 0.001
Ongoing pregnancies (n,%)	1505/5873 (25.63%)	65/414 (15.70%)	< 0.001
Live birth (n,% per embryo transfer cycle)	1463/5873 (24.92%)	59/414 (14.25%)	< 0.001
Live birth (n,% per clinical pregnancy)	1463/1801 (81.23%)	59/87 (67.82%)	0.003
Singleton (% per live birth)	1437/1463 (98.22%)	59/59 (100%)	0.622
Twins (% per live birth)	26/1463 (1.78%)	0/59 (0%)	
Ectopic pregnancy (n,%)	52/5873 (0.89%)	1/414 (0.24%)	0.260
Biochemical pregnancy losses (n,%)	201/2054 (9.79%)	14/102 (13.73%)	0.195
Early miscarriages (n, % per clinical pregnancy)	296/1801 (16.44%)	22/87 (25.29%)	0.039
Second trimester abortions (n, % per clinical pregnancy)	40/1801 (2.22%)	5/87 (5.75%)	0.053
Stillbirths (n, % per clinical pregnancy)	2/1801 (0.11%)	1/87 (1.45%)	0.132

Table 3 describes neonatal outcomes of singletons born after single FET with or without blastomere loss. All of the indicators listed were broadly comparable between the two groups. Specifically, the gestational age of the intact embryo group was similar to that of the blastomere loss group. There seemed to be no statistical difference in the mode of delivery or the sex of neonates between the groups. There was also no discernable evidence of a difference in birth weight, birth length, Z-scores adjusted for the gestational age and gender, and the incidence of SGA and LGA. Besides, a total of 1 neonate died from multiple organ failure in the neonatal period in the intact embryo group, while no case was observed in the blastomere loss group. Therefore, no difference was distinguished with respect to congenital defects or neonatal mortality between the two groups. Since there was no case of monozygotic twin in the blastomere loss group, we simply presented the neonatal outcomes of twins in the intact embryo group in Supplemental Table 1, in which the average gestational age and birthweight were 35.08 ± 2.49 weeks and 2377.08 ± 509.59 g, with 9.62% SGA and no case of congenital defects or neonatal mortality.

**Table 3:** Outcomes of singletons born after transfer embryos with or without blastomere loss

	Intact embryo group (*n* = 1437)	Blastomere loss group (*n* = 59)	*P*
Gestational Age (weeks)	38.40 ± 1.76	38.23 ± 1.70	0.598
Mode of delivery			0.666
Vaginal	437/1437 (30.41%)	16/59 (27.12%)	
Cesarean section	1000/1437 (69.59%)	43/59 (72.88%)	
Sex (female)	684/1437 (47.60%)	28/59 (47.46%)	0.971
Birthweight (g)	3278.04 ± 502.38	3328.17 ± 386.82	0.587
< 2500 g	90/1437 (6.26%)	2/59 (3.39%)	0.434
> 4000 g	88/1437 (6.12%)	2/59 (3.39%)	
Z-scores	−0.01 ± 0.99	0.29 ± 0.86	0.107
Birth length (cm)	49.85 ± 2.09	50.03 ± 1.19	0.626
Birthweight for gestational age			0.803
SGA	77/1437 (5.36%)	2/59 (3.39%)	
LGA	189/1437 (13.15%)	8/59 (13.56%)	
Congenital defects	27/1437 (1.88%)	2/59 (3.39%)	0.318
Neonatal mortality	1/1437 (0.07%)	0/59 (0%)	1.000

Table 4 displays the relationship between the percentage of blastomere loss and the pregnancy outcomes following single FET. There was a general trend that as the percentage of blastomere loss increased, the biochemical pregnancy rate, clinical pregnancy rate, ongoing pregnancy rate, live birth rate per embryo transfer cycle, and live birth rate per clinical pregnancy decreased remarkably. After further post hoc analysis, it was found that the differences of the above pregnancy-related indicators were significant regarding the comparison between the intact embryo group and the group with less than 25% blastomere loss and the comparison between the intact embryo group and the group with 26–50% blastomere loss, while the difference of the other pairwise comparison was undetected. Furthermore, it was revealed that the early miscarriage rate was comparable among the three groups with different percentages of blastomere loss.

**Table 4:** Pregnancy outcomes related to the percentage of blastomere loss

	Percentage of blastomere loss			*P*^1^	*P*^2^	*P*^3^	P*
	0% (*n* = 5873)	≤25% (*n* = 375)	26–50% (*n* = 39)				
Biochemical pregnancies (n,%)	2054/5873 (34.97%)	96/375 (25.60%)	6/39 (15.38%)	< 0.001	0.177	0.011	< 0.001
Clinical pregnancies (n,%)	1801/5873 (30.67%)	81/375 (21.60%)	6/39 (15.38%)	< 0.001	0.417	0.037	< 0.001
Ongoing pregnancies (n,%)	1505/5873 (25.63%)	61/375 (16.27%)	4/39 (10.26%)	< 0.001	0.487	0.027	< 0.001
Live births (n,% per embryo transfer cycle)	1463/5873 (24.92%)	55/375 (14.67%)	4/39 (10.26%)	< 0.001	0.631	0.039	< 0.001
Live births (n,% per clinical pregnancy)	1463/1801 (81.23%)	55/81 (67.90%)	4/6 (66.67%)	0.006	1.000	0.316	0.008
Early miscarriages (n, % per clinical pregnancy)	296/1801 (16.44%)	20/81 (24.69%)	2/6 (33.33%)	0.067	0.640	0.259	0.085

*P*^1^:1VS2

*P*^2^:2VS3

*P*^3^:1VS3

Table 5 reports the multiple binary logistic regression analyses results of factors that might have an impact on blastomere loss/live birth. For blastomere loss, primary infertility and ICSI were found to be common influencing factors. Primary infertility was demonstrated to be associated with an increase in the probability of blastomere loss, in comparison to secondary infertility (aOR 1.447, 95% CI 1.038–2.019, *P* = 0.029), while patients undergoing ICSI were 1.388 times more likely to suffer from blastomere loss (aOR: 1.388, 95% CI: 1.044–51.846, *P* = 0.024), compared with patients taking IVF as the insemination method. For live birth, the results turned out that the maternal age, the number of blastomere loss and the embryo quality were influencing factors. Patients with advanced maternal age and inferior embryo quality were less likely to have a live birth (aOR: 0.901, 95% CI: 0.882–0.921, *P* < 0.001; aOR: 0.753, 95% CI: 0.657–0.863, P < 0.001; respectively), while the number of blastomere loss was negatively associated with live birth (aOR: 0.655, 95% CI: 0.493–0.869, *P* = 0.003).

**Table 5:** Binary logistic regression on blastomere loss/live birth in patients undergoing FET (*n* = 6287)

Blastomere loss	aOR (95% CI)	*P*
Maternal age (years)	0.977 (0.934–1.023)	0.330
Parental age (years)	0.999 (0.962–1.039)	0.979
Maternal BMI (kg/m^2^)	0.992 (0.959–1.025)	0.627
Duration of infertility (years)	1.014 (0.970–1.060)	0.527
Primary infertility	1.447 (1.038–2.019)	0.029
Pluriparous	1.224 (0.776–1.931)	0.384
Insemination method (ICSI vs. IVF)	1.388 (1.044–1.846)	0.024
Blastomere number at cryopreservation	1.006 (0.871–1.163)	0.933
**Live birth**	aOR (95% CI)	*P*
Maternal age (years)	0.901 (0.882–0.921)	< 0.001
Parental age (years)	1.001 (0.983–1.019)	0.956
Maternal BMI (kg/m^2^)	0.995 (0.983–1.007)	0.376
Duration of infertility (years)	1.009 (0.987–1.032)	0.421
Primary infertility	0.888 (0.769–1.026)	0.108
Pluriparous	0.951 (0.768–1.178)	0.647
Insemination method (ICSI vs. IVF)	0.936 (0.864–1.025)	0.152
Number of blastomere loss	0.655 (0.493–0.869)	0.003
Blastomere number on embryo transfer	1.043 (0.979–1.112)	0.191
Embryo quality (grade 2 vs. grade 1)	0.753 (0.657–0.863)	< 0.001

**Fig. 1:**
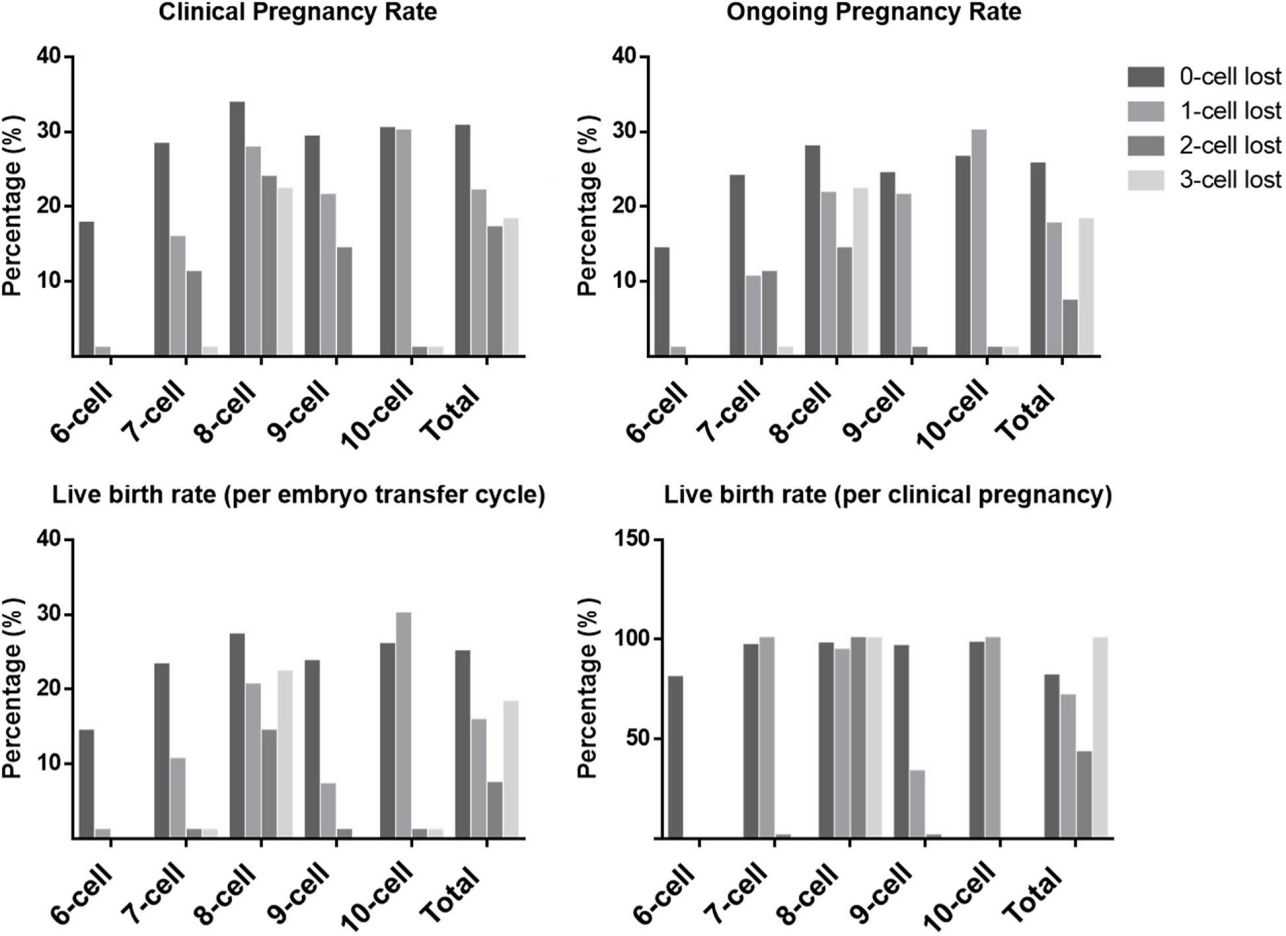
The pregnancy outcomes of cleavage-stage embryos with different number of blastomere loss following single FET

Figure 1 specifically exhibits the pregnancy outcomes of cleavage-stage embryos following single FET, according to cell stage at cryopreservation and the number of blastomere loss. In accordance with the findings presented in Table 2 and Table 4, similar trends were identified for each cell stage at cryopreservation, that with the increasing blastomere loss at thawing, the clinical pregnancy rate, ongoing pregnancy rate and live birth rate per cycle were progressively decreased. In particular, when it was a 6-cell embryo at cryopreservation, even the loss of only one blastomere would reduce the clinical pregnancy rate to zero; while when the cell number was 7 to 10 at cryopreservation, one blastomere loss at thawing would result in a reduction in less than 50% clinical pregnancy rate. Even more to the point, the clinical pregnancy rate of an 8-cell or 10-cell embryo with one blastomere loss at thawing was comparable to that of an intact embryo. As for the live birth rate per clinical pregnancy, it kept roughly the same as the number of blastomere loss increased. Take a closer look at the 8-cell embryo, the live birth rate per clinical pregnancy was 97.28% (787/809), 94.22% (17/18), 100% (3/3), 100% (2/2), when the number of blastomere loss was 0,1,2,3, respectively.

## Discussion

This retrospective cohort study of 6287 single FET cycles aimed to investigate the impact of blastomere loss of D3 vitrified embryos on embryonic developmental potentials and neonatal outcomes. The results showed that the embryos with blastomere loss presented with a lower implantation rate, biochemical/clinical/ongoing pregnancy rate, and live birth rate per embryo transfer cycle/per clinical pregnancy, compared with intact embryos. Binary logistic regression analysis further confirmed the negative influence of blastomere loss on live birth. Moreover, the early miscarriage rate of embryos with blastomere loss was higher. Additionally, the neonatal conditions were broadly similar between the neonates resulting from intact embryos and embryos with blastomere loss. Furthermore, primary infertility and ICSI were found to be related to an increased likelihood of blastomere loss.

The present study showed that the D3 embryos with blastomere loss had an obvious decreased developmental potential than intact embryos. This finding is mostly in agreement with a series of previous studies [13–19]. The reason for the lower viability of embryos with blastomere loss remains not clearly clarified to date. Possible speculations are made that damaged blastomeres may induce a detrimental/toxic effect on the other blastomeres [26]. In brief, blastomere loss is considered to be caused by the blastomere lysis induced by intracellular ice formation, hyperosmotic damage, and metabolic derangements. As blastomeres degrade, the lysed cells disrupt cell-to-cell communication and lose viable embryonic materials [27, 28].

Despite so many supportive studies, this issue is still full of controversy. Some studies declared that blastomere loss was not associated with an elevated miscarriage rate [17, 19]. Whereas in our study, the biochemical pregnancy rate, one of the embryonic factors putatively involved in the process of implantation [29], seemed to be higher in the blastomere loss groups (though without statistical significance). Moreover, the early miscarriage rate was significantly higher in the blastomere loss group, from which we could speculate that blastomere loss might have a negative effect on embryonic development throughout the first trimester, lasting even until the confirmation of clinical pregnancies. The difference between our results and the previous ones [17, 19] might be due to the difference in experimental design and patients’ enrollment, that is, we only included all the vitrified/warmed D3 cleavage-stage single frozen-thawed embryo transfer (FET) cycles as the research objects.

Besides, some studies declared that blastomere loss was conditionally not negatively correlated with implantation rate. Some studies found that when embryos were only transferred if they resumed mitotic activity after thawing, on the occasion of laser-assisted hatching and necrotic blastomeres removal, there was no adverse effect of blastomere loss on implantation potential [11, 12]. On the other hand, some studies reported that cell loss was completely irrelevant to a reduced embryo competence [8–10]. On closer interpretation, however, most of these studies focused on D2 embryos, which was different from the objective of our study [8, 9]. Another study limited its scope to 8-cell D3 embryos with 1 or 2 cells lost, the results of which could not be generalized [10]. The discrepancy in the results reported may be attributed to variability within the studies. Hence, our study provides a more robust result with a large sample size and a fixed setting of single FET of vitrified/warmed D3 embryos with different cell-stage and number of blastomere loss, while without additional auxiliary operations (i.e., assisted hatching, necrotic blastomeres removal, overnight culture).

When further dividing the embryos with blastomere loss into subgroups according to their percentage of blastomere loss, we revealed a general trend that the higher the percentage of blastomere loss, the lower the pregnancy rate and the live birth rate. Furthermore, even though the blastomere loss of an embryo was within 25%, its conception ability was still impaired. This result differs from reported literature, in which embryos were not vulnerable when blastomere loss was less than 25% [10, 17]. The reason for this discordance might come from the inclusion of a portion of slow freezing protocol in their studies [10, 17]. On the one hand, the survival rate of cleavage-stage embryos is apparently higher for embryos frozen by vitrification than by slow freezing (6.59% in our study by vitrification) [30, 31]. On the other hand, due to the defects of the slow freezing technique itself, even though the cell damage seems to be undetectable or minor morphologically, slow freezing will still have a negative impact on the subsequent development of embryos [6, 12]. Based on this premise, we can easily understand that for the embryos lost less than 25% of blastomeres, as the proportion of slow freezing increases, the pregnancy rate decreases. Therefore, the superiority of vitrification is fully embodied and we suggest vitrification as a better technique.

In addition, we took careful explorations on the pregnancy outcomes, according to cell stage at cryopreservation and the number of blastomere loss. Specifically, for embryos that lost one blastomere, the clinical pregnancy rate of 6-cell embryos would reduce to zero; the pregnancy outcomes of 8-cell or 10-cell embryos were comparable to those of intact embryos; the conception probability of 7-cell or 9-cell embryos was partially impaired. In cases where two blastomeres were lost, 8-cell embryos still maintained a clinical pregnancy rate of 23.81%, while 9-cell embryos dropped its rate to 14.29%. When the situation came to 3 blastomeres lost, only 8-cell embryos reserved a clinical pregnancy rate of 22.22%. These detailed results suggested that for different cell stage embryos, the same percentage of cell damage may have completely different effects on their developmental potential. Therefore, when encountering blastomere loss, clinicians should make decisions after careful considerations. For instance, when performing preimplantation genetic testing (PGT), we strongly recommend 8-cell embryos as the highest priority and avoid choosing 6-cell embryos. Another applicable situation was 6cell embryos with blastomere loss in single FET, in which we should consider warming one more embryo to compensate for the sharp reduction in clinical pregnancy rate.

As for the neonatal outcomes of singletons, it was observed that the two groups were comparable. These results are partially consistent with previous studies [17, 19]. While both Wu YT et al. and L.C. O’Shea et al. demonstrated no differences in birth weight in their study, the former declared an association between blastomere loss and SGA. The discrepancy might be interpreted by their inclusion of both slow freezing and vitrification cycles, considering the underlying harmful influence of slow freezing on the embryos. Our study, therefore, is more reassuring and addresses the long-term safety of blastomere loss regarding the neonatal outcomes, validating the safety of embryos with blastomere loss in vitrified/warmed cycles. Besides, since there were no twins in the blastomere loss group, we inferred that embryos with blastomere loss were less likely to develop into monozygotic twins.

In our study, primary infertility and ICSI were found to be risk factors for blastomere loss, a topic that has not been discussed previously. Primary infertility was usually recognized as an internal influencing factor. Previous studies demonstrated a lower pregnancy probability in patients diagnosed with primary infertility, compared with secondary infertile couples [32, 33], indicating the impaired developmental potential of patients with primary infertility and manifesting as blastomere loss embryologically. On the other hand, ICSI is perceived as an external influencing factor. To date, a number of studies have reported a reduced ability of ICSI-derived embryos to develop to the blastocyst stage [34, 35]. Since this phenomenon has also been reported in a sibling oocyte study [36], it is suggested that it might be related to the injection procedure rather than to the origin of the male gamete. Besides, the developmental rate of ICSI embryos are faster compared to IVF embryos (at least during 48 h) [37] and more advanced embryos (9–10-cell) may suffer from the detrimental freezing effect of mitotic cells [38]. Therefore, ICSI probably results in embryos with more cells when cryopreservation and subsequent blastomere loss when warming due to the damage of mitotic cells.

The present study has several salient strengths. First, it is the largest population study so far with a sample size of 5287, focusing on the impact of blastomere loss on the pregnancy outcomes of D3 vitrified cleavage-stage embryos in single FET cycles. Second, we calculate the concrete pregnancy rate according to cell stage at cryopreservation and the number of blastomere loss, in a detailed manner. These results provide valuable information for assessing the success rate and specific clinical guidance for the corresponding strategy. Moreover, we are among the pioneers investigating the neonatal outcomes of newborns originating from embryos with blastomere loss. Our findings confirm the safety of embryos with blastomere loss in vitrified/warmed cycles and offer the obstetricians and pediatricians reassuring conclusions. In addition, we take the lead in exploring the risk factor of blastomere loss. The creative results remind clinicians to strictly mater the indications of ICSI and manipulate prudently in the procedure, especially for the high-risk populations with primary infertility.

However, our results also have some limitations. First, due to the low incidence of birth defects and neonatal mortality, these variables have wide CIs. Second, only patients who completed their followup were enrolled, though the overall follow-up rate in our center was around 98%. Thus, the intentionto-treat principle was not fully followed. Third, since whether embryos were compacted at the time of freezing was absent in our record, we failed to explore whether compaction contributed to blastomere loss, although only a very small proportion of embryos developed to the compacted state upon freezing. Last, this was a retrospective study, although the study was strictly executed according to good clinical practice guidelines. Hence, a large-sample prospective trial is necessary in the future.

## Conclusion

In conclusion, the D3 embryos with blastomere loss induced by vitrification/warming show impaired developmental potential in terms of implantation rate, biochemical pregnancy rate, clinical pregnancy rate, ongoing pregnancy rate, live birth rate per embryo transfer cycle, and live birth rate per clinical pregnancy, as well as an elevated early miscarriage rate. Moreover, advanced maternal age, inferior embryo quality and increased blastomere loss are turned out to be negatively correlated with live birth. Of note, even the loss of less than 25% of blastomeres may be detrimental. The detailed analysis demonstrates that the impact of 1 blastomere loss for 8-cell or 10-cell embryos is negligible, while for 6-cell embryos is fatal, which provides clinicians with more specific guidance when making decisions on embryo selection. Moreover, the neonatal conditions of embryos derived from blastomere loss embryos and intact embryos are comparable and blastomere loss is not associated with an increased risk of any adverse neonatal outcomes in the singletons, preliminarily confirming the safety of transferring embryos with blastomere loss. Additionally, primary infertility and ICSI are proven to be risk factors for blastomere loss, which can be applied for predicting the occurrence as well as reducing the incidence of blastomere loss. Long-term follow-up studies with larger a sample size and prospective design are necessary to be carried out for investigating the possible effects on child growth and development.

## Supplementary Information

**Additional file 1.**

## References

[1] Zeilmaker GH, Alberda AT, van Gent I, Rijkmans CM, Drogendijk AC (1984). Two pregnancies following transfer of intact frozen-thawed embryos. Fertil Steril.

[2] Wong KM, Mastenbroek S, Repping S (2014). Cryopreservation of human embryos and its contribution to in vitro fertilization success rates. Fertil Steril.

[3] Thurin A, Hausken J, Hillensjö T, Jablonowska B, Pinborg A, Strandell A (2004). Elective singleembryo transfer versus double-embryo transfer in in vitro fertilization. N Engl J Med.

[4] Zech J, Brandao A, Zech M, Lugger K, Neururer S, Ulmer H (2018). Elective frozen-thawed embryo transfer (FET) in women at risk for ovarian hyperstimulation syndrome. Reprod Biol.

[5] Loutradi KE, Kolibianakis EM, Venetis CA, Papanikolaou EG, Pados G, Bontis I (2008). Cryopreservation of human embryos by vitrification or slow freezing: a systematic review and meta-analysis. Fertil Steril.

[6] Debrock S, Peeraer K, Fernandez Gallardo E, De Neubourg D, Spiessens C, D'Hooghe TM (2015). Vitrification of cleavage stage day 3 embryos results in higher live birth rates than conventional slow freezing: a RCT. Hum Reprod.

[7] Veleva Z, Orava M, Nuojua-Huttunen S, Tapanainen JS, Martikainen H (2013). Factors affecting the outcome of frozen-thawed embryo transfer. Hum Reprod.

[8] Edgar DH, Archer J, McBain J, Bourne H (2007). Embryonic factors affecting outcome from single cryopreserved embryo transfer. Reprod BioMed Online.

[9] Gabrielsen A, Fedder J, Agerholm I (2006). Parameters predicting the implantation rate of thawed IVF/ICSI embryos: a retrospective study. Reprod BioMed Online.

[10] Zheng X, Liu P, Chen G, Qiao J, Wu Y, Fan M (2008). Viability of frozen-thawed human embryos with one–two blastomeres lysis. J Assist Reprod Genet.

[11] Rienzi L, Ubaldi F, Iacobelli M, Giuliaminasi M, Romano S, Ferrero S (2005). Developmental potential of fully intact and partially damaged cryopreserved embryos after laser-assisted removal of necrotic blastomeres and post-thaw culture selection. Fertil Steril.

[12] Van Landuyt L, Van de Velde H, De Vos A, Haentjens P, Blockeel C, Tournaye H (2013). Influence of cell loss after vitrification or slow-freezing on further in vitro development and implantation of human day 3 embryos. Hum Reprod.

[13] Tang R, Catt J, Howlett D (2006). Towards defining parameters for a successful single embryo transfer in frozen cycles. Hum Reprod.

[14] El-Toukhy T (2003). Effect of blastomere loss on the outcome of frozen embryo replacement cycles. Fertil Steril.

[15] Guerif F, Bidault R, Cadoret V, Couet ML, Lansac J, Royere D (2002). Parameters guiding selection of best embryos for transfer after cryopreservation: a reappraisal. Hum Reprod.

[16] Edgar DH, Bourne H, Speirs AL, McBain JC (2000). A quantitative analysis of the impact of cryopreservation on the implantation potential of human early cleavage stage embryos. Hum Reprod.

[17] Wu Y-T, Li C, Zhu Y-M, Zou S-H, Wu Q-F, Wang L-P (2018). Outcomes of neonates born following transfers of frozen-thawed cleavage-stage embryos with blastomere loss: a prospective, multicenter, cohort study. BMC Med..

[18] Capodanno F, De Feo G, Gizzo S, Nicoli A, Palomba S, La Sala GB (2016). Embryo quality before and after slow freezing: viability, implantation and pregnancy rates in 627 single frozen-thawed embryo replacement cycles following failure of fresh transfer. Reprod Biol.

[19] O'Shea LC, Hughes C, Kirkham C, Mocanu EV (2016). The impact of blastomere survival rates on developmental competence of cryo-thawed day 2 embryos. Eur J Obstet Gynecol Reprod Biol.

[20] Kuang Y, Chen Q, Fu Y, Wang Y, Hong Q, Lyu Q (2015). Medroxyprogesterone acetate is an effective oral alternative for preventing premature luteinizing hormone surges in women undergoing controlled ovarian hyperstimulation for in vitro fertilization. Fertil Steril.

[21] Yu S, Long H, Chang HY, Liu Y, Gao H, Zhu J (2018). New application of dydrogesterone as a part of a progestin-primed ovarian stimulation protocol for IVF: a randomized controlled trial including 516 first IVF/ICSI cycles. Hum Reprod.

[22] Zhang J, Mao X, Wang Y, Chen Q, Lu X, Hong Q (2017). Neonatal outcomes and congenital malformations in children born after human menopausal gonadotropin and medroxyprogesterone acetate treatment cycles. Arch Gynecol Obstet.

[23] Huang J, Xie Q, Lin J, Lu X, Wang N, Gao H (2019). Neonatal outcomes and congenital malformations in children born after dydrogesterone application in progestin-primed ovarian stimulation protocol for IVF: a retrospective cohort study. Drug Des Devel Ther.

[24] Meersseman W, Verschueren P, Tousseyn T, De Vos R, Cassiman D. PAS-positive macrophages—not always infection. Lancet. 2011;377(9780):1890. 10.1016/S0140-6736(11)60285-7 21621718

[25] Denning PW, Dai L, Deng C, Li Y, Zhu J, Mu Y, et al. Birth weight reference percentiles for Chinese. Plos One. 2014;9(8):e104779. 10.1371/journal.pone.0104779 PMC4134219 25127131

[26] Dulioust E, Toyama K, Busnel MC, Moutier R, Carlier M, Marchaland C (1995). Long-term effects of embryo freezing in mice. Proc Natl Acad Sci U S A.

[27] Elliott TA, Colturato LF, Taylor TH, Wright G, Kort HI, Nagy ZP (2007). Lysed cell removal promotes frozen-thawed embryo development. Fertil Steril.

[28] Liu WX, Zheng Y, Luo MJ, Huang P, Yue LM, Wang L (2005). Effects of removal of necrotic blastomeres from mouse cryopreserved embryos on blastocyst formation and hatching. Theriogenology.

[29] Vaiarelli A, Cimadomo D, Patrizio P, Venturella R, Orlando G, Soscia D (2018). Biochemical pregnancy loss after frozen embryo transfer seems independent of embryo developmental stage and chromosomal status. Reprod BioMed Online.

[30] Rezazadeh Valojerdi M, Eftekhari-Yazdi P, Karimian L, Hassani F, Movaghar B (2009). Vitrification versus slow freezing gives excellent survival, post warming embryo morphology and pregnancy outcomes for human cleaved embryos. J Assist Reprod Genet.

[31] Rienzi L, Gracia C, Maggiulli R, LaBarbera AR, Kaser DJ, Ubaldi FM (2017). Oocyte, embryo and blastocyst cryopreservation in ART: systematic review and meta-analysis comparing slow-freezing versus vitrification to produce evidence for the development of global guidance. Hum Reprod Update.

[32] Templeton A, Morris JK, Parslow W (1996). Factors that affect outcome of in-vitro fertilisation treatment. Lancet.

[33] Stolwijk AM, Wetzels AM, Braat DD (2000). Cumulative probability of achieving an ongoing pregnancy after in-vitro fertilization and intracytoplasmic sperm injection according to a woman's age, subfertility diagnosis and primary or secondary subfertility. Hum Reprod.

[34] Miller JE, Smith TT (2001). The effect of intracytoplasmic sperm injection and semen parameters on blastocyst development in vitro. Hum Reprod.

[35] Archer J (2003). Blastocyst formation and cell numbers in human frozen-thawed embryos following extended culture. Hum Reprod.

[36] Griffiths TA, Murdoch AP, Herbert M (2000). Embryonic development in vitro is compromised by the ICSI procedure. Hum Reprod.

[37] Dumoulin JC, Coonen E, Bras M, van Wissen LC, Ignoul-Vanvuchelen R, Bergers-Jansen JM (2000). Comparison of in-vitro development of embryos originating from either conventional invitro fertilization or intracytoplasmic sperm injection. Hum Reprod.

[38] Chedid S, Van den Abbeel E, Van Steirteghem AC (1992). Effects of cryopreservation on survival and development of interphase- and mitotic-stage 1-cell mouse embryos. Hum Reprod.

