# Supplementary material for: The impact of blastomere loss on pregnancy and neonatal outcomes of vitrified-warmed Day3 embryos in single embryo transfer cycles
Source: J Ovarian Res. 2022 May 18;15:62. doi: 10.1186/s13048-022-00997-z (PMC9116052; doi:10.1186/s13048-022-00997-z)
Supplement: Supplementary file 1 — Additional file 1. [file 13048_2022_997_MOESM1_ESM.docx]

Supplemental Table 1

Neonatal outcomes of monozygotic twins born after transfer of intact embryos

|  | Intact embryo group (n=52) |
| --- | --- |
| Gestational Age (weeks) | 35.08±2.49 |
| Mode of delivery |  |
| Vaginal | 2/52 (3.85%) |
| Cesarean section | 50/52 (96.15%) |
| Sex (female) | 26/52(50.00%) |
| Birthweight (g) | 2377.08±509.59 |
| <2500g | 22/52 (42.31%) |
| >4000g | 0/52 (0%) |
| Birth length (cm) | 46.52±3.45 |
| Birthweight for gestational age |  |
| SGA | 5/52 (9.62%) |
| LGA | 0/52 (0%) |
| Congenital defects | 0/1437 (0%) |
| Neonatal mortality | 0/52 (0%) |
